# A Case of Bladder Perforation With Rectal Impalement Injury

**DOI:** 10.1002/iju5.70101

**Published:** 2025-10-07

**Authors:** Tatsuma Juichi, Atsushi Fujikawa

**Affiliations:** ^1^ Department of Urology Yokosuka City Hospital Yokosuka Japan

**Keywords:** bladder perforation, rectal impalement, transurethral coagulation

## Abstract

**Introduction:**

Combined rectal impalement (RI) and bladder perforation (BP) is an extremely rare injury pattern, with limited case reports and no established consensus on their diagnosis and management.

**Case Presentation:**

A 50‐year‐old man sustained a perianal impalement injury caused by a metal rod at a construction site. He presented with perianal pain and gross hematuria (GH). Imaging revealed RI and extraperitoneal BP. A colostomy was performed on the same day as the injury, and transurethral coagulation of the bladder (TUC) was performed on the 6th day, during which a bladder mucosal defect was identified. Postoperatively, the GH reduced, and no persistent voiding or defecation dysfunction was observed.

**Conclusion:**

In patients with RI and GH, concomitant BP should be suspected. Although colostomy is almost always required for rectal injury, extraperitoneal BP can be cured with transurethral intervention and catheterization.

**Summary**

- Simultaneous rectal impalement and bladder perforation is extremely rare.
- Gross hematuria with confirmed rectal injury should raise suspicion of bladder involvement.
- Transurethral surgery and urethral catheterization were effective for extraperitoneal bladder injury.

**Abbreviations**
BP: bladder perforation
CE: contrast enema
CT: computed tomography
eGFR: estimated glomerular filtration rate
RI: rectal impalement
TUC: transurethral coagulation of bladder
UC: urethral catheter

## 1 Introduction

The combination of rectal impalement (RI) and bladder perforation (BP) was first described over 50 years prior [1]. Despite this, reported cases remain rare [2]. Therefore, continued accumulation of case reports and individualized, case‐by‐case clinical approaches are essential. Here, we present a rare case of combined RI and BP that was successfully treated using a multidisciplinary approach.

## 2 Case Report

A 50‐year‐old man with a history of cerebral hemorrhage in his 20s and residual right hemiparesis, although ambulatory without assistive devices, experienced trauma at a construction site. He fell backward onto an 8‐cm fixed metal rod protruding from the floor, which penetrated his perianal region. He managed to stand and detach from the rod, which remained embedded in the floor, but continued to experience lower abdominal and perineal pain, along with gross hematuria (GH). He presented to the emergency department approximately 2 h post‐injury.

**FIGURE 1:**
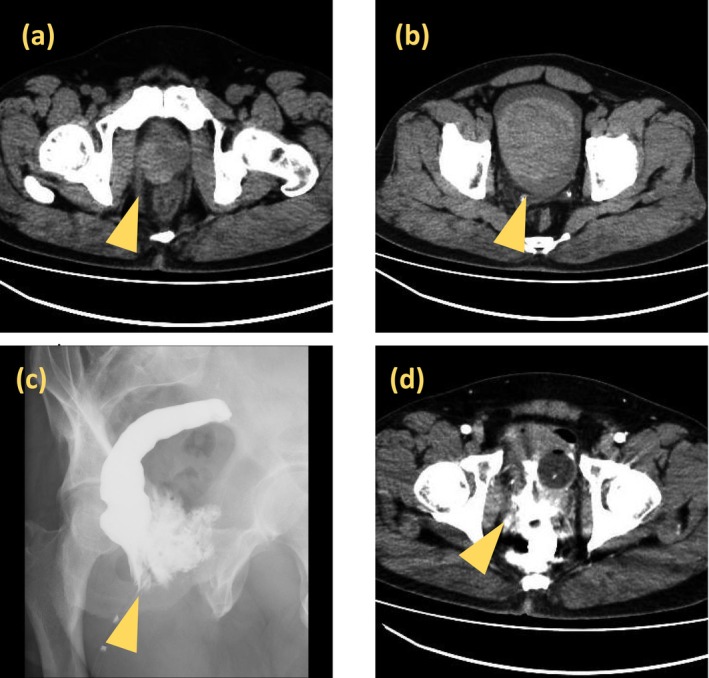
(a) Non‐contrast CT showing a high‐density area (approximately 4 cm) between the right anterior wall of the rectum and the right dorsolateral prostate, suggestive of hematoma. (b) CT revealing a high‐density area (approximately 10 cm) within the bladder, also indicative of hematoma. (c) CE demonstrating extravasation of contrast medium outside the rectal lumen without bladder visualization. (d) Post‐enema CT showing high‐density contrast accumulation around the rectum, prostate, bladder, and perivesical space, raising suspicion of combined rectal and bladder injury without evidence of intraperitoneal leakage.

Physical examination revealed a 2‐cm depressed perianal wound at the 11 o'clock position without active bleeding. Continuous venous bleeding from the external urethral meatus and a strong sensation of urinary retention were observed. Laboratory tests revealed mild anemia (Hb 11.4 g/dL) and elevated levels of inflammatory markers. Computed tomography (CT) showed a 4‐cm hematoma between the right anterior rectal wall and right dorsolateral prostate (Figure 1a) and a 10‐cm hematoma in the bladder (Figure 1b). Fat stranding was also observed around the rectum, particularly on the right side. No foreign bodies or free air were detected. Contrast enema (CE) revealed extravasation outside the rectum without communication with the bladder or urethra (Figure 1c). Post‐CE CT showed contrast accumulation in the rectum, perirectal space, bladder, and perivesical area, indicating combined RI and BP (Figure 1d); however, no leakage into the peritoneal cavity was observed.

A urethral catheter (UC) was then inserted. The GH was mild but persistent despite saline irrigation, raising the suspicion of BP.

**FIGURE 2:**
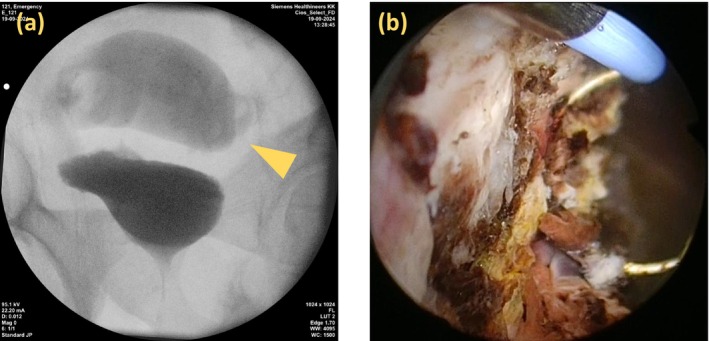
(a) Retrograde urethrocystography showing extravasation of the contrast medium outside the bladder with suspected opacification of the sigmoid colon or rectum. (b) Endoscopic image revealing a mucosal defect on the right lateral bladder wall with exposed perivesical fat, following removal of a 2‐cm hematoma. This site was identified as the source of the hematuria.

Emergency management prioritized the rectal injury, and a diverting colostomy was performed on the same day. During surgery, 150 mL of indigo carmine‐dyed saline was instilled into the bladder; no peritoneal leakage was observed. After the diverting colostomy, the GH gradually reduced, but did not fully resolve. On hospital day 6, transurethral surgery was performed with the patient under general anesthesia. During the procedure, simultaneous retrograde urethrocystography demonstrated contrast leakage outside the bladder with suspected visualization of the sigmoid colon or rectum (Figure 2a). Endoscopy revealed no urethral damage; however, a 2‐cm hematoma was found on the right bladder wall lateral to the right ureteral orifice. After removal, a mucosal defect with exposed fat was observed (Figure 2b), which was determined to be the BP. Coagulation achieved hemostasis, and no other bladder injuries were identified. A urethral catheter was re‐inserted postoperatively. Following the procedure, the GH was nearly resolved.

One month later, CE revealed no rectal leakage. The patient continued to undergo outpatient follow‐ups with the UC in place. No further GH or urinary leakage was observed. Three months postoperatively, cystoscopic evaluation demonstrated a mildly ulcerative lesion at the previous site of the BP; however, the perforation itself was confirmed to be fully closed. Simultaneous contrast imaging revealed no evidence of contrast medium leakage, and the UC was subsequently removed. Colostomy closure was completed 6 months post‐injury. A follow‐up cystoscopy at 7 months revealed scarring at the injury site; however, the lesion had reduced in size. The patient had no voiding dysfunction or recurrence of GH.

## 3 Discussion

RI injuries are typically caused by penetrating trauma from foreign objects entering directly or via the perineum and may lead to intraperitoneal or extraperitoneal rupture of the rectum and other organs, most commonly within the genitourinary tract. Although isolated RI or BP have been frequently reported, secondary BP following RI is extremely rare. Benjelloun et al. compiled 14 such cases [2], and Harada et al. subsequently reported an additional case [3]. Among the 15 cases referenced above, 13 were male, with a median age of 34 years (range: 9–57 years), indicating a predominance of younger men.

GH is the most frequent symptom of BP, occurring in up to 95% of the cases [4]. Because rectal injury can be relatively easily diagnosed through digital rectal examination and CE, the presence of GH in a patient with a confirmed rectal injury, such as in the present case, should prompt a strong suspicion of concomitant BP.

The European Association of Urology recommends the use of CT cystography to obtain rapid diagnostic information, particularly when there is a possibility of concomitant abdominal trauma [5]. In the present case, CT was performed after CE on the day of the injury, and a diverting colostomy was performed based on the diagnosis of rectal injury. The diagnosis of BP was later confirmed using TUC combined with cystography. Additionally, indigo carmine was instilled intraoperatively into the bladder to rule out intraperitoneal perforations. Although prioritizing CT cystography is reasonable in cases of isolated BP, in cases such as the present case where multiple organ injuries are involved, the diagnostic and therapeutic priorities must be carefully considered on a case‐by‐case basis.

In cases of combined rectal and bladder perforating trauma, surgical management typically involves diverting colostomies for rectal injury, and treatment outcomes are generally favorable [2, 3]. Because the most feared complication in similar cases is peritonitis due to gastrointestinal perforation, thorough evaluation of gastrointestinal lesions is essential in managing combined RI and BP. In this case, gastrointestinal surgical intervention performed on the day of injury was the greatest success factor in the treatment strategy. This prompt intervention likely contributed to the favorable clinical outcome.

When managing bladder injury, treatment planning depends on the type of injury, making it essential to distinguish between intraperitoneal and extraperitoneal BP. Intraperitoneal BP usually requires immediate surgical repair, whereas extraperitoneal BP is often managed conservatively with urethral catheterization for several weeks and has a good prognosis [6]. Extraperitoneal BP with RI is typically managed using urethral catheterization and diverting colostomy [2].

## 4 Conclusion

Simultaneous injuries to the rectum and bladder are rare. However, this case underscores the importance of considering concurrent bladder injury in patients presenting with RI and GH. Accurate diagnosis requires a dual approach: CE for rectal evaluation and cystography for bladder assessment. Although diverting colostomy is almost always necessary, the management of BP depends on whether the perforation is intraperitoneal or extraperitoneal. In cases involving extraperitoneal bladder injury, such as the present one, TUC for hematuria control combined with urethral catheterization has been shown to yield favorable treatment outcomes.

## Consent

The patient provided informed consent for publication of this case report.

## Conflicts of Interest

The authors declare no conflicts of interest.
